# Idiopathic Ventricular Arrhythmias Originating from the Left Ventricular Summit: A Diagnostic and Therapeutic Challenge

**DOI:** 10.3390/jcm14124261

**Published:** 2025-06-16

**Authors:** Raffaele Falco, Chiara Tognola, Lorenzo Gigli, Matteo Baroni, Antonio Frontera, Marisa Varrenti, Alberto Preda, Marco Carbonaro, Roberto Menè, Leandro Fabrizio Milillo, Andrea Sultana, Sara Vargiu, Giulia Colombo, Federica Giordano, Cristina Giannattasio, Patrizio Mazzone, Fabrizio Guarracini

**Affiliations:** 1 Electrophysiology Unit, De Gasperis Cardio Center, Niguarda Hospital, 20162 Milan, Italy; lorenzo.gigli@ospedaleniguarda.it (L.G.); matteo.baroni@ospedaleniguarda.it (M.B.); antonio.frontera@ospedaleniguarda.it (A.F.); marisa.varrenti@ospedaleniguarda.it (M.V.); alberto.preda@ospedaleniguarda.it (A.P.); marco.carbonaro@ospedaleniguarda.it (M.C.); roberto.mene@ospedaleniguarda.it (R.M.); leandrofabrizio.milillo@ospedaleniguarda.it (L.F.M.); andrea.sultana@ospedaleniguarda.it (A.S.); sara.vargiu@ospedaleniguarda.it (S.V.); giulia.colombo@ospedaleniguarda.it (G.C.); federica.giordano@ospedaleniguarda.it (F.G.); patrizio.mazzone@ospedaleniguarda.it (P.M.); fabrizio.guarracini@ospedaleniguarda.it (F.G.); 2 Clinical Cardiology Unit, De Gasperis Cardio Center, Niguarda Hospital, 20162 Milan, Italy; chiara.tognola@ospedaleniguarda.it (C.T.); cristina.giannattasio@ospedaleniguarda.it (C.G.)

**Keywords:** ventricular premature complexes, arrhythmias, cardiac, catheter ablation

## Abstract

Premature ventricular contractions (PVCs) originating from the left ventricular summit (LVS) present a diagnostic and therapeutic challenge due to their complex anatomical location. The LVS includes an epicardial area of the left ventricle bordered by major coronary arteries, which has been increasingly recognized as an arrhythmic focus. Idiopathic ventricular arrhythmias from this area may exhibit specific electrocardiographic characteristics, making accurate localization essential for effective management. **Methods**: This narrative review explores the primary features of this arrhythmia, emphasizing key diagnostic and therapeutic aspects, including both pharmacological and interventional approaches, considering the recent technological advances in cardiac mapping and ablations. **Conclusions**: PVCs originating from the left ventricular summit (LVS) exhibit characteristic electrocardiographic features. Prompt recognition of this arrhythmia may facilitate appropriate referral for targeted treatment.

## 1. Introduction

PVCs originating from the LVS are a noteworthy clinical entity due to their distinctive electrophysiological properties and associated clinical implications. Despite their characteristic features, this arrhythmia remains underrecognized and often underdiagnosed. Pharmacological therapy represents the first-line approach in management, but ablation remains a cornerstone, particularly in cases of pharmacological failure and PVC-induced cardiomyopathy. A detailed understanding of the PVC presentation, LVS anatomy, and its relationships with other cardiac structures is essential for mapping and ablating LVS arrhythmias.

## 2. The Aims and the Methods of the Review

The aim of our work is to present a narrative review highlighting the main diagnostic challenges associated with this arrhythmia, while providing clinicians with the tools necessary for its recognition and optimal management, particularly in light of recent advances in cardiac ablation techniques. We conducted a comprehensive literature search using major scientific databases, including PubMed, Embase, and the Cochrane Central Register of Controlled Trials. The search strategy employed the following combined keywords: Ventricular Premature Complexes, Arrhythmias, Tachycardia, and Catheter Ablation. In addition, a manual search of reference lists and citations was performed to identify further relevant publications. Most of the studies identified were single-center trials and case reports.

## 3. Anatomy of LVS

The LVS represents a triangular epicardial region of the left ventricle, bordered superiorly by the bifurcation of the left main (LM), which forms its apex, and is located between the anterior descending coronary artery (LAD) and the circumflex artery (LCx). The triangle’s base is formed by an imaginary curved line, with a radius corresponding to the distance from the apex to the origin of the first prominent left coronary artery septal perforator from the LAD, directed towards the LCx. The surface of the LV summit is traversed by the anterior interventricular vein (AIV), which runs along the anterior interventricular sulcus before joining the posterior interventricular sulcus and becoming the great cardiac vein (GCV). The AIV divides the LVS into an upper and a lower region. The first is called the Brocq and Mouchet triangle, and it is considered inaccessible for an interventional approach due to the high risk of life-threatening complications arising from the presence of coronary vessels and abundant adipose tissue. The lower region, however, is considered a safer area for intervention, accessible via endocardial and epicardial approaches or even through the coronary sinus or the left atrial appendage [1]. The average dimensions of the LVS, measured using specific contrast-enhanced CT protocols, are approximately 263 mm^2^, with the upper, inaccessible area being larger than the lower area (133 vs. 95 mm^2^) [2]. Furthermore, Yamada proposed a formula that, by considering the angle of the left main bifurcation (LM) and the dimensions of the septal and mitral margins, as evaluated by coronary angiography, can reliably estimate the dimensions of the LVS (mean value of 291.58 mm^2^) [3]. Moreover, approximately 75% of the LVS (196.08 mm^2^) can covered by the left atrial appendage along the mitral margin, with the “windsock” morphology being the most common type, associated with the highest degree of LVS coverage (80%) [2,4].

The LVS can have anatomical variants due to abnormalities in the arterial and venous coronary circulation. Based on the intersection of the GCV with the LAD and LCx, five anatomical variants of the Brocq and Mouchet triangle have been described [5].

## 4. Incidence

The exact incidence of PVCs from the LV summit remains challenging to define due to underdiagnosis and difficulties in correctly identifying their origin. However, several studies have focused on their frequency in both general and special populations, reporting a variable incidence ranging between 15% and 20% of all idiopathic PVCs [6,7,8]. They are typically observed in middle-aged and healthy individuals, but can also occur, less frequently, in patients with underlying structural heart disease. No gender differences have been highlighted across studies, although a slight predominance in men has been seen. In high-volume ablation centers, about 10–20% of patients undergoing PVC ablation present the main focus in LVS [9,10].

## 5. Clinical Presentation

PVCs originating from LVS present with a variety of clinical manifestations, ranging from asymptomatic patients to invalidating symptoms, depending on PVC burden and underlying structural heart disease. Symptoms more frequently occur when the PVC burden exceeds 10–20% or when arrhythmia occurs in bigeminal or trigeminal patterns. The main symptoms are palpitations, exercise intolerance, and PVC-induced cardiomyopathy [3,11] (Table 1).

Palpitations are reported to be more frequent in idiopathic PVCs from LVS compared to those originating from other outflow tracts [12]. The fatigue and exercise intolerance are linked to ventricular dyssynchrony, which leads to a suboptimal cardiac output during physical activity [9]. Although rare, atypical chest pain has also been reported. This pain is generally non-ischemic and may be related to the mechanical effects of frequent ventricular contractions. Approximately 10–15% of patients with PVCs from LVS report episodes of sharp and brief chest pain [13].

The most severe clinical presentation is PVC-induced cardiomyopathy, closely connected to a daily PVC burden greater than 20%. Some studies report an LV dysfunction risk of 12–15% during a period of 1–2 years [14]. This finding is crucial in identifying the need for early intervention, even in asymptomatic cases [9]. In rare cases, in susceptible individuals, particularly in those with underlying structural heart disease, PVCs from LVS can be a trigger for more malignant arrhythmias and they are associated with ventricular tachycardia (VT) or ventricular fibrillation (VF).

Furthermore, a significant proportion of patients may remain asymptomatic, without reducing the potential risk arrhythmia-induced cardiomyopathy or the development of more malignant arrhythmias. Bogun et al. report that asymptomatic patients with a PVC burden exceeding 10,000 per day are at a higher risk of developing PVC-induced cardiomyopathy [13]. Moreover, nearly 25% of patients with frequent PVCs from the LV summit exhibited subclinical reductions in the left ventricular ejection fraction despite having no overt symptoms.

## 6. Diagnostic Evaluation

### 6.1. 12-Lead ECG

The origin of PVCs from LVS can be assessed with a 12-lead ECG evaluation. Notably, PVCs may exhibit a right bundle branch block (RBBB) pattern with an inferior axis, characterized by larger R waves in lead III compared to lead II, or a left bundle branch block (LBBB) pattern with an inferior axis and rapid precordial transition.

**Figure 1:**
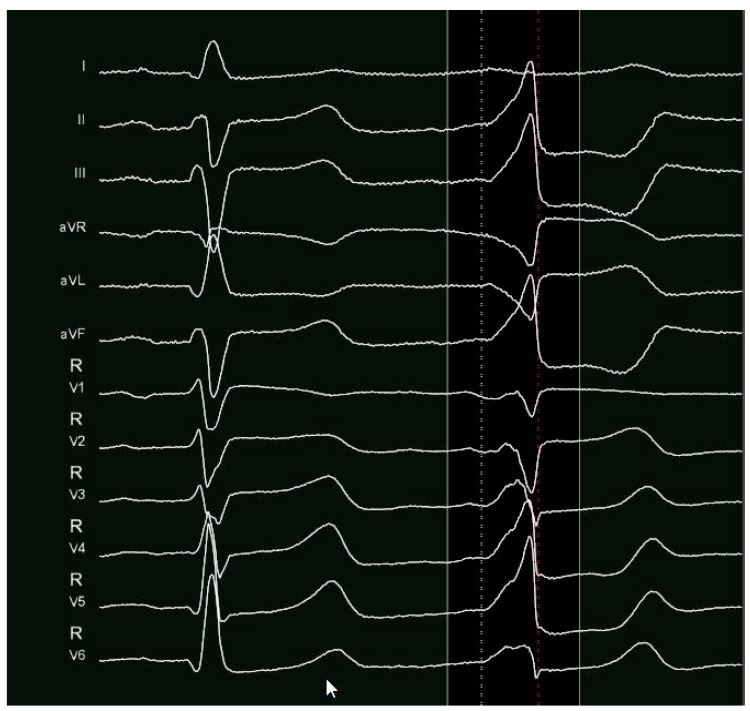
PVC from LV summit. An LBBB pattern with an inferior axis and a rapid precordial transition (V3). The “pseudodelta wave” and the MDI > 0.54 suggest an epicardial origin.

Several ECG findings can predict the epicardial origin, reflecting a delayed initial activation of the LV: a time to earliest rapid deflection in precordial leads (pseudodelta wave) longer than 34 ms; an interval to the peak of the R wave in lead V2 (intrinsicoid deflection time—IDT) greater than 85 ms; a time to the earliest QRS nadir in precordial leads (shortest RS complex) longer than 121 ms; and a maximum deflection index (MDI) greater than 0.54 [15]. The MDI can be calculated as a ratio between the time to the maximum deflection interval to the peak of QRS and the QRS duration (Figure 1).

Moreover, the presence or absence of a Q wave can reflect local epicardial ventricular activation: for example, a Q wave in lead I correlates with a superior epicardial origin site and can be present in 30% of cases. Another particular ECG feature is the “breakthrough pattern” in lead V2, characterized by the sudden disappearance of the R wave in lead V2, followed by its recovery in lead V3. This suggests a septal origin of LVS because it is anatomically opposed to lead V2 and it identifies a PVC hard to ablate because it is usually close to the LAD, so in the inaccessible area [15,16].

Other ECG findings can be useful for planning the site of ablation (endocardial or epicardial) and for stratifying the procedural success rate. This depends on the possible origin of the PVC from the inaccessible area of the LV. The Q-wave ratio in leads aVL/aVR may predict with high sensitivity and specificity the most probable site of ablation: this ratio is around 1.41 to 1.53 in cases of the subaortic valve region, while it is around 1.53 to 1.74 in cases of the GCV/AIV vein junction. If values exceed 1.74, epicardial treatment is suggested, and higher values significantly correlate with an origin from the accessible LVS area [17]. Additionally, an RBBB pattern or LBBB with early precordial transition and S waves in lead V5 or V6 is typically observed in patients with a PVC originating in the accessible area. Instead, PVCs from the inaccessible region of the LVS usually exhibit a higher R amplitude in inferior leads [16].

Finally, it is also possible to identify patients with a higher likelihood of successful epicardial ablation. The presence of at least two of the following criteria is associated with successful epicardial ablation with 100% sensitivity and 72% specificity: a Q-wave ratio in leads aVL/aVR > 1.85, R/S wave ratio in lead V1 > 2, and lack of initial Q wave in lead V1 [18]. Another study demonstrated that a “w” sign in lead I in combination with an early precordial pattern break and a maximum deflection index (MDI) ≥ 0.5 had sensitivity and specificity for a successful ablation in the distal GCV of 94.4% and 96.1%, respectively [19].

### 6.2. Imaging

The precise localization and characterization of PVCs originating from the LV summit is crucial for effective treatment planning, especially when considering catheter ablation. Advanced imaging modalities, particularly cardiac magnetic resonance imaging (MRI) and 3D electroanatomical mapping (EAM), have revolutionized the ability to identify PVC foci and guide treatment decisions [20].

- *Cardiac MRI*

Cardiac MRI allows for structural abnormalities’ and fibrotic changes’ evaluation, which can be essential in differentiating idiopathic PVCs from those secondary to underlying heart disease. The role of MRI in PVC localization and arrhythmia mapping has been extensively documented in the clinical literature [21,22]. In patients with frequent PVCs from the LV summit, some authors reported that MRI frequently reveals fibrotic spots near the aortic root and LV outflow tract. Presence of myocardial scarring is associated with a higher risk of developing VT and PVC-induced cardiomyopathy. In patients with a high PVC burden, MRI can identify early signs of ventricular remodeling and dilatation, even before significant clinical symptoms develop. Cardiac MRI is particularly useful in patients with PVC-induced cardiomyopathy, where imaging may show regional contractile dysfunction in areas adjacent to the PVC origin [21,23]. Finally, cardiac MRI offers the advantage of assessing ventricular function, helping to quantify the impact of frequent PVCs on the left ventricular ejection fraction (LVEF).

- *Intracardiac Echocardiography (ICE)*

Considering the anatomical complexity of the LVS region, ICE imaging can be employed to guide the electroanatomic mapping by assessing the position and contact of the ablation catheter with the target structures and reducing the use of fluoroscopy [24]. When the ICE probe is in the right atrium, counterclockwise rotation allows for visualization of the RVOT, while clockwise rotation reveals the aortic valve plane and ascending aorta in a long-axis view. To obtain a short-axis view of the aortic valve plane, it is necessary to advance the probe into the right ventricle while applying a slight clockwise rotation. Some mapping systems can process echocardiographic images acquired via the ICE probe to create a detailed 3D shell for subsequent ablation (Soundstar ICE, CARTOSOUND, Biosense Webster). This allows for the reconstruction of the anatomical area of the pulmonary and aortic valve planes, as well as the ostia and course of the coronary arteries [25].

## 7. Pharmacological Treatment

Various classes of antiarrhythmic drugs can be employed in order to reduce the PVC burden and prevent progression to PVC-induced cardiomyopathy. Given the variability in patient response to medical treatment, a combination therapy is often employed in clinical practice (Table 2).

- *Beta-blockers*

Beta-blockers are often the first-line treatment for managing LV summit PVCs, especially in symptomatic patients or those who present with a high PVC burden [26]. Their antiarrhythmic effect is primarily achieved by inhibiting sympathetic activity, thereby reducing the excitability of the myocardium and the likelihood of PVC occurrence. Extensive evidence supports the use of beta-blockers in managing both idiopathic and non-idiopathic PVCs, above all when structural heart disease is present [27,28]. Metoprolol and bisoprolol have been shown to significantly reduce the PVC burden and improve quality of life in patients with LV summit PVCs, with reports of symptomatic improvement. However, their efficacy in reducing the PVC burden is variable, and some patients may not experience sufficient symptom relief or PVC reduction with beta-blockers alone, requiring adjunctive therapies to achieve optimal control [20,26].

- *Sodium channel blockers*

Class I antiarrhythmic drugs can offer an alternative when beta-blockers are insufficient or not tolerated. Flecainide is highly effective in patients with idiopathic PVCs, compared to sotalol and propafenone [29]. In a recent trial, flecainide showed a significantly greater reduction in PVC burden compared with metoprolol in a pediatric population without structural heart disease [30]. Furthermore, in patients suspected of having PVC-induced cardiomyopathy, class I-AADs effectively suppressed PVCs, leading to LVEF recovery in the majority of patients [31]. Propafenone can also be a therapeutic option, although its beta-blocking properties make it less desirable in patients who are intolerant of beta-blockers. In terms of safety, the use of Class I agents is known to be contraindicated in patients with structural heart disease, given the raised arrhythmic risk [32].

- *Potassium channel blockers*

Class III antiarrhythmic agents, such as amiodarone and sotalol, represent a second-line therapy when beta-blockers and Class I agents fail to control symptoms or when PVCs occur in the setting of underlying cardiac disease. Amiodarone has demonstrated efficacy in reducing the PVC burden in patients with frequent and symptomatic PVCs, including those arising from the LV summit, both in structural and non-structural heart disease [33]. Amiodarone was also effective in reducing the PVC frequency and improving the exercise capacity in patients with PVC-induced cardiomyopathy [34].

Sotalol can be used in patients with LV summit PVCs. While its efficacy is less established than that of amiodarone, sotalol has shown moderate success in patients with structurally normal hearts who are intolerant of beta-blockers or sodium channel blockers. Sotalol reduces with high efficacy the PVC burden in patients with idiopathic PVCs, although its use is limited by the potential for QT interval prolongation and torsades de pointes [35].

- *Calcium Channel Blockers*

Non-dihydropyridine calcium channel blockers (CCBs) may be considered an alternative when previous pharmacological treatments have been ineffective. Studies regarding CCB efficacy in LV summit PVC suppression are lacking.

## 8. Interventional Treatment

The LVS represents a challenging region to ablate, even for expert operators, due to the intricate anatomic relationship involving different endocardial and epicardial regions. LVS can be directly accessed via percutaneous epicardial puncture, but more often it is targeted from endocardial-adjacent structures, due to the high procedural risk of an epicardial ablation. Arrhythmias originating from the LVS can be treated from the coronary venous system, and when the origin is suspected to be intramural below the epicardial LVS, they can also be ablated from the left coronary cusp (LCC), LVOT endocardium, or septal RVOT. Some cases report ablations from the left atrial appendage.

**Table 2:** Pharmacological treatment.

Drug Class	Mechanism	Efficacy	Limitations
Beta-blockers	Sympathetic activity	PVC burden; symptoms; useful in structural heart disease	Fatigue, bradycardia, and hypotension
Sodium Channel Blockers	Block sodium channels	PVC burden: symptoms and quality of life	Proarrhythmic risk in structural heart disease
Potassium Channel Blockers	Prolonged action potential and refractory period	PVC burden; exercise capacity in PVC-induced cardiomyopathy	Thyroid and pulmonary toxicity; QT prolongation
Calcium Channel Blockers	Inhibit calcium influx	Effective in some cases of idiopathic PVCs	Limited evidence base
Combination Therapy	Combines mechanisms to achieve better symptom control	Useful when monotherapy is insufficient	Increased risk of side effects; requires close monitoring

- *Endocardial Ablation*

This is typically the first-line approach for LV summit PVC ablation, due to its relatively lower risk and more straightforward access compared to epicardial techniques. Through the endocardial approach and the electroanatomic mapping systems, it is possible to reach and ablate the PVC focus via the left ventricle, the left ventricular outflow tract (LVOT), the RVOT, the aortic cusps, or other nearby endocardial regions. However, the anatomical limitations of the LV summit present significant challenges. Some areas are not directly accessible endocardially, particularly those adjacent to the coronary arteries. Furthermore, careful attention is required to avoid damage to critical structures such as the aortic valve, coronary arteries, and the His–Purkinje system. The septal RVOT should be the first endocardial site of mapping, above all if the 12 lead-ECG suggests an ROVT origin (left bundle branch block pattern and transition after V3 with an inferior axis), also considering that in many patients, septal endocardial RVOT and AIV can be very close. When approaching septal RVOT, it is important to advance the catheter as leftward as possible in an LAO fluoroscopic view. If ablation is performed in this location, it is essential to first identify and avoid the pulmonary valve plane, due to its anatomical proximity to the inaccessible region of the LVS and the left main. A retrograde arterial approach is required for LCC and LVOT mapping. ICE can assist in catheter localization, obtaining a short-axis image of the aortic cusp region [36]. To enhance procedural success, additional techniques such as pacing maneuvers and detailed 12-lead ECG analysis are often employed to confirm the proximity of the ablation catheter to the PVC origin. Intracardiac echocardiography is also a valuable tool for assessing catheter–tissue contact and minimizing procedural risks. Subsequently, the ablation catheter can be advanced across the aortic valve to map the LV subvalvular endocardium below the LCC. An LAO fluoroscopic view can be useful for catheter tip orientation, while the location can be achieved using the ICE [10,16]. Potential complications of an endocardial strategy mainly include aortic valve damage (mechanical catheter trauma or RF delivery to the valvular tissue) and embolic events (thrombus or air). An irrigated catheter and accurate periprocedural anticoagulation can limit these complications.

For PVCs originating from epicardial or deeper myocardial layers, endocardial ablation may be insufficient, requiring alternative strategies [18,37].

- *Coronary Sinus Ablation*

**Figure 2:**
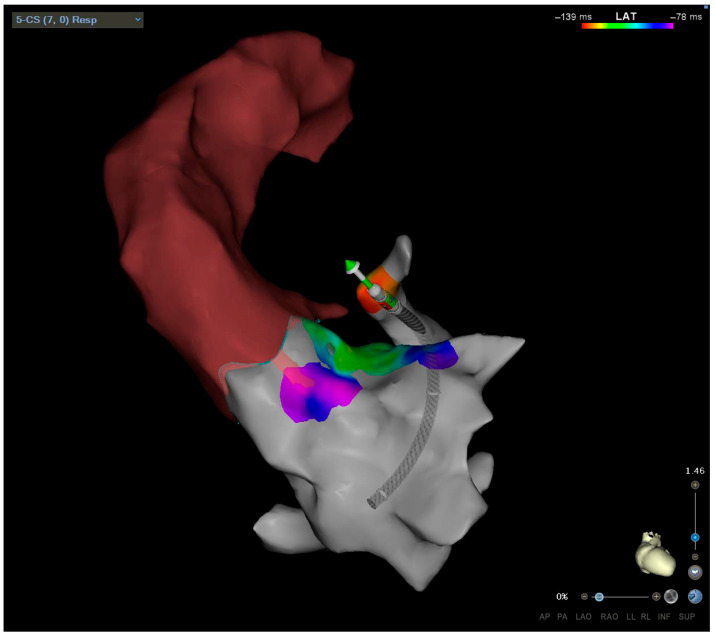
LVS approach through coronary sinus.

An epicardial etiology of arrhythmias is postulated if the GCV/AIV activation time is earlier than that of the other RVOT and LVOT locations. The coronary venous system provides an alternative pathway for ablation when endocardial techniques fail (Figure 2). The intramural component of the LV septum can be mapped after selective cannulation of a septal venous perforator branch, guided by a venogram of the AIV branches [38]. An epicardial LVS origin is suggested by a ventricular activation time at the distal GCV or proximal AIV earlier than in other previous sites mapped, the LVOT, LCC, or RVOT, usually with a good pace–map match. An earlier ventricular activation time in GCV/AIV is typical of epicardial VT/PVCs, while an earlier activation time in the first septal perforator suggests an intramural PVC focus. Precise mapping and careful catheter manipulation are critical to achieving successful outcomes [39]. The main limitations are challenging cannulation of target venous vessels, inability to achieve adequate power because of impedance or temperature rise, and, finally, proximity to coronary vessels. A coronary angiogram is not necessary if ICE clearly shows a safe distance from the ablation catheter tip to the LM [40]. RF energy must be delivered carefully using irrigated-tip catheters with a step-wise incremental power from 20 W to 40 W, a temperature limit of 42 °C, and an impedance decrease of 10 Ω to 15 Ω, to prevent overheating and minimize tissue damage [16]. However, the intramural origin and the proximity to coronary vessels can limit RF success ablation. Ablation from the coronary sinus may result in acute artery vessel injury and thrombosis requiring immediate intervention, or vein perforation resulting in pericardial effusion to cardiac tamponade.

- *Ethanol Ablation*

Ethanol ablation can be an alternative when PVC remains refractory to RF ablation. This can be possible when the arrhythmogenic focus has a deep midmyocardial or an epicardial origin, and thus RF energy cannot reach the focus with a sufficient therapeutic effect [41]. Ethanol is a water-soluble compound that at high concentrations can rapidly cross and solubilize cell membranes, leading to immediate cell destruction, and its infusion intramural branches allow for reaching the epicardial and intramural arrhythmogenic focus. Ethanol infusion can be performed via the coronary arteries (anterograde) or through the coronary sinus (retrograde). Although, in a large series of RF-refractory VTs, Kumar demonstrated the efficacy of transcoronary ethanol ablation, where the risk of atrioventricular block is high [42]. Furthermore, potential complications include coronary arterial dissection, thrombosis, and myocardial infarction. The venous approach is associated with a lower procedural risk. The target branch of the coronary sinus can be identified using small multipolar catheters or with an angioplasty wire configured as a unipolar electrode. Once early pre-systolic activity has been documented, ethanol can be delivered using balloon inflation to counteract venous flow, thereby preventing ethanol leakage into the coronary sinus or collateral vessels. Ethanol is injected over one minute, followed by repeat contrast administration to assess myocardial staining. Repeated ethanol injections can be performed to achieve PVC elimination. However, ethanol ablation is a challenging procedure due to the technical difficulty and the anatomical variability of the coronary sinus. A study on a small cohort of patients with RF-resistant PVCs demonstrates its efficacy and low risks [43,44].

- *Left Atrial Appendage*

The left atrial appendage (LAA) is an area of growing interest for the ablation of PVCs originating from the LVS, given its anatomical location. It partially covers the mitral margin of the LVS to varying degrees depending on its morphology [2]. Preoperative computed tomography assessment can evaluate the dimensions, morphology, and relationships between the LVS and LAA for optimal procedural planning. Only a few case reports describe this approach as a final attempt for endocardial ablation after the failure of all other approaches, before considering an epicardial approach [45]. The earliest activation signal is sought in the LAA using a transseptal approach. RF energy is delivered using irrigated catheters, with progressively increasing power up to a maximum of 40 W for 40 s [46]. Due to the possibility of LAA perforation, RFA from LAA is often avoided.

- *Epicardial Ablation*

In individuals whose endocardial ablation via the nearby LVS regions is ineffective, epicardial mapping and ablation may be attempted. This approach targets the arrhythmogenic substrate directly from the epicardial surface, but the ablation is complex because of the closeness of the major epicardial coronary arteries and the high quantity of epicardial fat in this area. Close mapping and angiographic imaging are essential to identify the course and ostium of the coronary arteries and the distance from catheter ablation at the earliest location. Additionally, the potential for phrenic nerve damage and diaphragmatic paralysis necessitates careful pacing at a high output to delineate the course and avoid nerve injury [47]. Other risks include pericardial complications, such as effusion and tamponade, which require prompt recognition and management. In approximately two-thirds of cases, RFA cannot be performed. However, the efficacy of the epicardial method in LVS VA ablation is low, with an acute success rate of around 22.0% and a long-term arrhythmia suppression rate of 17.0% [48]. Cryoablation has been employed as an alternative to RF in isolated cases [49].

- *Surgical Ablation*

Surgical ablation has been successfully employed in rare cases of refractory PVC-induced cardiomyopathy originating from the LVS. In one example, endocardial mapping through the RVOT, LVOT, and aortic sinus of Valsalva, combined with epicardial mapping via the great cardiac vein, failed to identify a suitable target. A percutaneous epicardial approach was also deemed unsuitable due to the proximity of the coronary arteries. The patient, who also required coronary artery bypass, underwent the procedure in a hybrid operating room. During the surgery, CARTO 3D mapping was used to identify the arrhythmogenic site. Cryoablation was then performed effectively eliminating PVCs [50]. However, surgical ablation is a complex option reserved for highly selected, refractory cases.

## 9. Conclusions

In summary, PVCs from the LV summit pose a complex clinical challenge due to their impact on patient symptoms, diagnostic intricacies, and treatment options. Understanding the incidence, diagnostic techniques, and management strategies for these arrhythmias is crucial for optimizing patient care. Continued research and technological advancements hold promise for further improving the diagnosis and treatment of LV summit PVCs, ultimately enhancing patient outcomes.

## Figures and Tables

**Table 1:** Clinical presentation.

Symptom	Notes
Palpitations	Common with high PVC burden, often linked to anxiety and exercise activity.
Fatigue	Caused by ventricular dyssynchrony and reduced cardiac output during activity.
Exercise Intolerance	Common in active individuals, linked to reduced cardiac output during exertion.
Chest Pain (Atypical)	Rare, sharp, non-ischemic pain from mechanical effects of PVCs.
PVC-Induced Cardiomyopathy	Risk increases with high PVC burden; untreated cases show progressive LV dysfunction.
Malignant Arrhythmias	Rare, but includes VT or VF, especially with structural heart disease or critical anatomy.
Asymptomatic	Common; a high PVC burden may still lead to cardiomyopathy or subclinical LV dysfunction.
